# The natural compound Sanggenon C inhibits PRRSV infection by regulating the TRAF2/NF-κB signalling pathway

**DOI:** 10.1186/s13567-023-01245-y

**Published:** 2023-11-30

**Authors:** Xiao Liu, Yanan Zhu, Dan Wang, Ran Feng, Zhihao Chen, Zifang Zheng, Yang Li, Lele Xu, Haixue Zheng, Yunpeng Fan, Yupeng Yin, Shuqi Xiao

**Affiliations:** 1 College of Veterinary Medicine, Northwest A&F University, Yangling, 712100 Shaanxi China; 2 State Key Laboratory for Animal Disease Control and Prevention, College of Veterinary Medicine, Lanzhou Veterinary Research Institute, Chinese Academy of Agricultural Sciences, Lanzhou University, Lanzhou, 730000 Gansu China

**Keywords:** PRRSV, natural compounds, Sanggenon C, TRAF2, NF-κB

## Abstract

Porcine reproductive and respiratory syndrome (PRRS) is a serious infectious disease and one of the major causes of death in the global pig industry. PRRS virus (PRRSV) strains have complex and diverse genetic characteristics and cross-protection between strains is low, which complicates vaccine selection; thus, the current vaccination strategy has been greatly compromised. Therefore, it is necessary to identify effective natural compounds for the clinical treatment of PRRS. A small molecule library composed of 720 natural compounds was screened in vitro, and we found that Sanggenon C (SC) was amongst the most effective natural compound inhibitors of PRRSV infection. Compared with ribavirin, SC more significantly inhibited PRRSV infection at both the gene and protein levels and reduced the viral titres and levels of protein expression and inflammatory cytokine secretion to more effectively protect cells from PRRSV infection and damage. Mechanistically, SC inhibits activation of the NF-κB signalling pathway by promoting TRAF2 expression, thereby reducing PRRSV replication. In conclusion, by screening natural compounds, we found that SC suppresses PRRSV infection by regulating the TRAF2/NF-κB signalling pathway. This study contributes to a deeper understanding of the therapeutic targets and pathogenesis of PRRSV infection. More importantly, our results demonstrate that SC has potential as a candidate for the treatment of PRRS.

## Introduction

Porcine reproductive and respiratory syndrome (PRRS) is one of the most harmful diseases in the pig industry. It can lead to reproductive disorders in pregnant sows and respiratory syndrome in weaned piglets and causes serious economic losses and great pressure in terms of disease prevention and control in the pig industry worldwide [1]. Since 2006, PRRS virus (PRRSV) has been widely prevalent in swine populations in China, causing immeasurable economic losses [2]. PRRSV infection costs approximately $664 million annually in the United States [3]. In Spain, the average loss due to PRRS infection was $200 per sow and $17.7 per piglet [4]. Because of PRRSV infection, Germany lost €31 per pig each year, and the average profits of PRRSV-positive farms decreased by 19.1% [5]. Korean economic losses were $7.50 per fattening pig and $204.3 per sow in pig farms with PRRS outbreaks [1].

Vaccination is currently considered to be the most competent approach to prevent PRRS. However, due to the rapid mutation of PRRSV strains, the relative lags in vaccine research and development, and the frequent recombination of different strains [6], the existing vaccines on the market can only provide limited protection and cannot completely prevent pigs from PRRSV infection [7]. Moreover, the continuous emergence of new strains, low cross-protection between strains, and restored or even enhanced virulence of attenuated vaccines by adaptation to the host and recombination add layers of difficulty in controlling PRRS [8]. These elusive strains of PRRSV greatly hinder the prevention and control of PRRS in the pig production industry and complicate the selection of vaccines and the formulation of vaccination strategies. Furthermore, the abuse of antibiotics in the pig industry poses an enormous threat to the ecological environment, food safety, and public health [9]. Therefore, there is an urgent need to find nonantibiotic natural compounds to prevent and treat PRRS efficiently and provide a new strategy for controlling PRRS in clinical practice.

In this study, 720 natural compounds were screened, and Sanggenon C (SC) was found to be a potential medicine against PRRSV infection. SC is a flavanone Diels–Alder adduct extracted from the root bark of plants in the mulberry genus that has blood pressure reducing, antiatherosclerotic, antioxidative, and anti-inflammatory effects [10] and thus the potential to be used as a natural medicine for a variety of diseases. Further studies have revealed that its potential antiviral mechanism is achieved by regulating the TRAF2/NF-κB signalling pathway. These results indicate that SC has potential as a candidate for the treatment of PRRS.

## Materials and methods

### Cell lines, viruses, and compounds

Porcine alveolar macrophages (PAMs) were isolated from 4- to 6-week-old PRRSV-free piglets [11] and cultured in RPMI 1640 medium (Gibco, Thermo Fisher Scientific, Inc.) containing 10% FBS. Marc-145, CRL-2843, Vero, PK-15, and HEK-293T cells were cultured in DMEM (Gibco, Thermo Fisher Scientific, Inc.) supplemented with 10% FBS. All cells were grown at 37 °C with 5% CO_2_. PRRSV (SD-YL1712) was amplified in Marc-145 cells. The PEDV, PCV2, CSFV, and PRV strains used in this study were isolated and preserved in our laboratory (Northwest A&F University, Shaanxi, China). Sanggenon C was purchased from TargetMol (USA), and ribavirin was purchased from MedChemExpress (USA).

### Antibodies and reagents

Anti-PEDV N and anti-PCV2 Cap polyclonal antibodies and anti-CSFV E2 and anti-PRRSV N monoclonal antibodies were prepared and stored in our laboratory (Northwest A&F University, Shaanxi, China). Anti-β-actin, anti-HA, anti-Flag, and anti-GFP monoclonal antibodies were obtained from TransGen Biotech. The anti-TRAF2 (A19129) monoclonal antibody was obtained from ABclonal Technology. The anti-p-TRAF2 (13908), anti-P65 (8242), anti-p-P65 (3033), anti-IκBα (9242), and anti-p-IκBα (5209) antibodies were purchased from Cell Signaling Technology. Lipofectamine™ RNAiMAX Transfection Reagent and Lipofectamine™ 3000 Transfection Reagent were obtained from Thermo Fisher Scientific. siRNA was synthesized by Tsingke Biotechnology.

### Cytotoxicity assay

Marc-145 cells, Vero cells and PK-15 cells were cultured in 96-well plates for 12 h, and PAMs were cultured in 96-well plates for 6 h. The cells were treated with different concentrations of SC, with 3 replicate samples of each concentration. After 36 h of treatment, cell proliferation was determined by CCK-8 assay to evaluate the cytotoxicity of SC. The experimental data were calculated using GraphPad Prism 8.

### Direct interaction between SC and PRRSV

To investigate whether SC can interact with PRRSV (10^6.23^ TCID_50_/mL) directly and decrease the viral titre, 10 µL of 1 mM SC solution was added to 990 µL of the original PRRSV solution to determine whether 10 µM SC could kill the virus. Moreover, 10 µL of DMSO was added to the negative control group, and the positive control group was treated with 10 µL neutralization antibody (1:64). After incubation at 4 °C for 1 h, cells were infected with 2 µL of virus mixture, and the supernatant and cell samples were collected at 36 and 48 hpi to detect the viral titres and PRRSV ORF7 and N protein expression levels.

### Immunofluorescence assays

IFAs were performed as described previously [11] with minor modifications. Cells were fixed with 4% paraformaldehyde for 20 min, permeabilized with 0.25% Triton X-100 at room temperature for 15 min, blocked with 2% bovine serum albumin (BSA) at room temperature for 1 h, rinsed with PBS three times, and incubated with mouse anti-Flag and goat anti-HA monoclonal antibodies at room temperature for 2 h. After washing with PBS, the cells were incubated with the corresponding fluorescent secondary antibodies for 1 h at room temperature. After staining with DAPI, the cells were visualized with a Nikon A1 confocal microscope.

### Western blotting and Co-IP

Western blotting was performed as described previously [12] with minor modifications. Cell samples were collected, protease and phosphatase inhibitors were added, and the cells were lysed for 20 min on ice. Then, the samples were centrifuged at 4 °C and 13 000 × *g* for 10 min, and the supernatant was collected to quantify the protein contents with a BCA kit. Loading buffer was added to boiling water for 10 min. The protein was isolated using a 12% SDS-PAGE gel and transferred to a PVDF membrane. The membrane was blocked in 5% skim milk at room temperature for 1 h, and the primary antibody was added for incubation at 4 °C overnight. The membrane was washed with PBST 3 times and incubated with a secondary antibody at room temperature for 1 h. The membrane was washed with PBST and incubated with ECL developing solution (Beyotime) to detect the target protein.

Magnetic beads were pre-treated with PBST. As described above, the supernatant was extracted from the lysed cells and added to anti-Flag or anti-HA magnetic beads (MCE). After incubation for 3 h at room temperature, PBST was used to wash the samples 5 times. Then, the protein samples were prepared for Western blot analysis.

### Quantitative reverse transcription PCR (RT-qPCR)

Total RNA was extracted from cells or the culture supernatant using TRIzol, cDNA was obtained with a PrimeScript RT kit, and then RT-qPCR analysis was performed with ChamQ SYBR qPCR Master Mix (Vazyme, Nanjing, China). The relative expression levels were calculated by the 2^−ΔΔCT^ method. The primers are listed in Table 1.

**Table 1:** **List of primers used in the RT-qPCR analysis**

Primers	Sequence (5′→3′)
pTNF-αF pTNF-αR pIL-1β-F	CCAGACCAAGGTCAACCTCC TCCCAGGTAGATGGGTTCGT ACCTGGACCTTGGTTCTCTG
pIL-1β-R	CATCTGCCTGATGCTCTTGT
pIL-8-F	AGAGTGGACCCCACTGTGAA
pIL-8-R	TGTACAACCTTCTGCACCCA
pIL-10-F	GAGATGATCCAGTTTTACCTGG
pIL-10-R pCXCL8-F pCXCL8-R pCXCL10-F pCXCL10-R pIL-17-F pIL-17-R pGAPDH-F pGAPDH-R	GATGACAGCGCCGCAGCCT AAAACCCATTCTCCGTGGCT GCAGCCTAGGGTTGCAAGAT ATAAGGATGGGCCGGAGAGA GTGGGAGCAGCTAACTTGGT ACAAAGTCCAGGATGCCCAA GGTGAGGTGAAGCGTTTGGA AGCAACAGGGTGGTGGACCT CTGGGATGGAAACTGGAAGT
PRRSV-ORF7-F	AATGGCCAGCCAGTCAATCA
PRRSV-ORF7-R	TCATGCTGAGGGTGATGCTG
mβ-actin-F	TCCCTGGAGAAGAGCTACGA
mβ-actin-R	AGCACTGTGTTGGCGTACAG

### Viral titration

A 50% tissue culture infective dose (TCID_50_) assay was performed to assess viral titration as described previously [13] with minor modifications. Marc-145 cells were seeded into 96-well plates 12 h prior to the experiments. The virus supernatant was prepared by 10-fold continuous dilution, and 100 µL of each dilution was added to each well with 8 replicate wells. The CPE numbers were determined after 4 days of culture, and TCID_50_ values were calculated by the Reed–Muench method.

### siRNA and plasmid transfection

Marc-145 cells were seeded in 12-well plates and transfected with siTRAF2 or siNC (negative control) at a dose of 50 nmol using Lipofectamine™ RNAiMAX Transfection Reagent (Thermo Fisher Scientific, CA, USA). The overexpression plasmid pXJ40-HA-TRAF2 and vector were transfected with Lipofectamine™ 3000 Transfection Reagent. Cells were collected at different time points for Western blotting.

### Time of treatment assay

SC was added before (pre-treatment), during (co-treatment), or after (post-treatment) PRRSV infection. The pre-treatment groups were inoculated with PRRSV (MOI = 1) 8, 6, 4, or 2 h after the addition of SC (10 µM). The co-treatment group was infected with PRRSV and immediately treated with SC. In the post-treatment group, cells were inoculated with PRRSV first, and SC was added at 2, 4, 6, or 8 h after infection. At 24 hpi, supernatant and cell samples were collected to detect viral titres and PRRSV ORF7 and N protein expression levels.

### Statistical analysis

All experiments were repeated three times and statistically analysed using GraphPad Prism 8 software. The results are expressed as the mean ± standard deviation (SD). Statistical significance was calculated by Student’s *t* test, and *P* values of *<* 0.05 (*), *<* 0.01 (**), and *<* 0.001 (***) were considered to indicate different levels of statistical significance. Image density was quantified using ImageJ software and normalized to β-actin.

## Results

### SC significantly inhibits PRRSV replication

**Figure 1:**
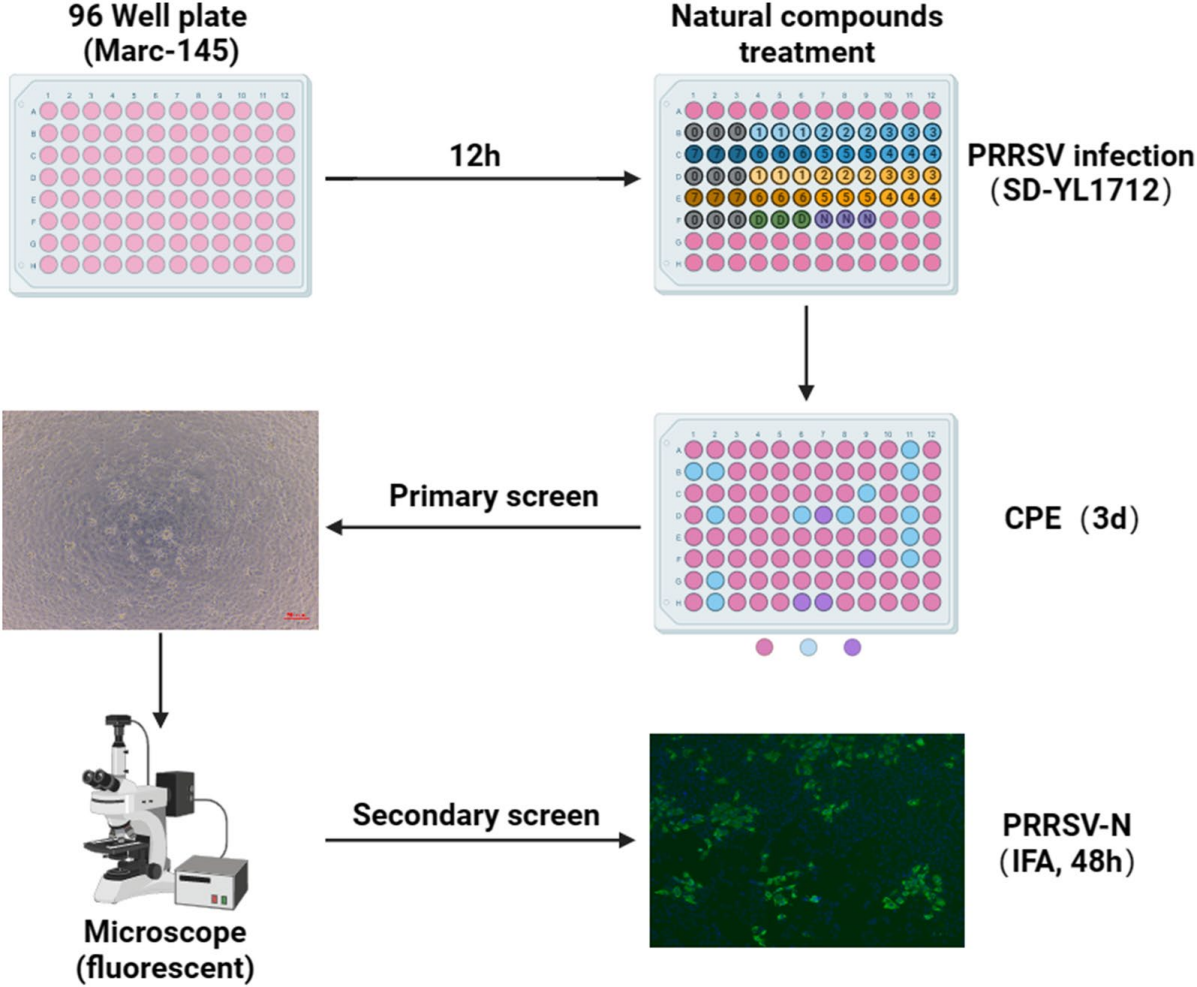
**Schematic diagram of the experimental design for screening natural compounds against PRRSV infection.**

Small molecule compound screening has been widely used to identify inhibitors and medicines to be used in potential prevention and treatment strategies for various viral infections, such as severe acute respiratory syndrome coronavirus 2 [14, 15], influenza A virus [16], dengue virus [17], and hepatitis C virus [18] infections. To identify natural molecules with inhibitory effects on PRRSV infection, Marc-145 cells were cultured in 96-well plates for 12 h, infected with PRRSV (MOI = 1) and then treated with small molecule natural compounds at a concentration of 10 µM. The cytopathic effect (CPE) was continuously monitored until 72 h post-infection (hpi) and considered to be the inhibitory effect of each compound; moreover, the effective compounds were screened repeatedly (Figure 1). Among the 720 tested natural compounds, Sanggenon C stood out for its ability to substantially block CPE production and viral replication, indicating that it could significantly inhibit PRRSV infection.

**Figure 2:**
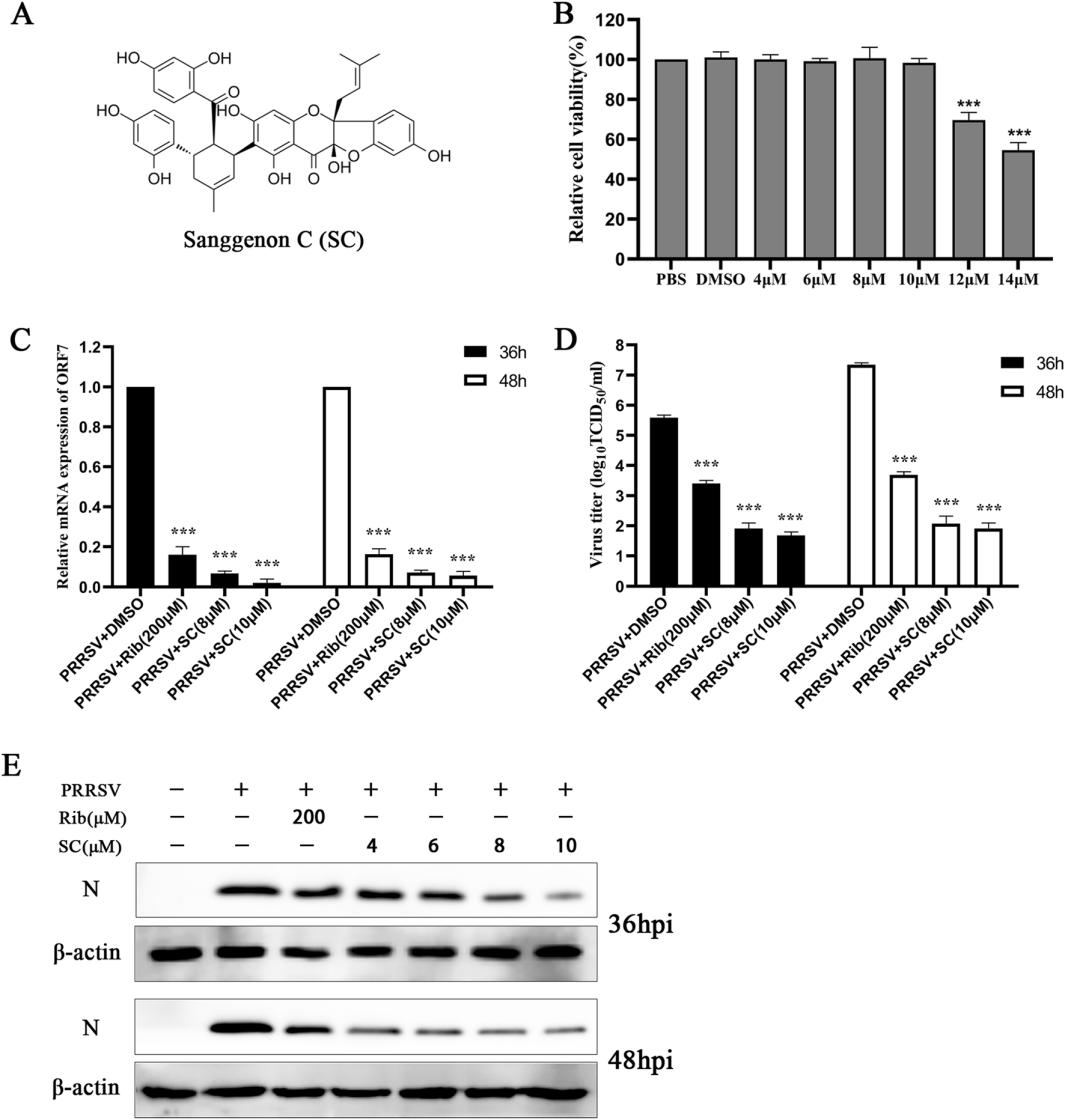
**SC significantly inhibits PRRSV replication.** **A** Chemical structure of Sanggenon C. **B** After 48 h of SC treatment, the toxicity of SC to Marc-145 cells was detected by CCK-8 assay. PBS was used as a negative control, and the cell viability in this group was set at 100%. **C**–**E** Marc-145 cells were seeded in 12-well plates, cultured for 12 h, infected with PRRSV SD-YL1712 (MOI = 1), and then treated with different concentrations of SC. Supernatants and cells were collected at 36 and 48 hpi to detect PRRSV ORF7 expression levels, virus titres and PRRSV N expression levels. *P < 0.05; **P < 0.01; ***P < 0.001 compared to the respective virus control.

We first evaluated the cytotoxicity of SC (Figure 2A) in Marc-145 cells using a CCK-8 assay. When the SC concentration was ≤ 10 µM, there was no significant effect for up to 48 h of culture (Figure 2B). Next, Marc-145 cells were infected with PRRSV (MOI = 1), and supernatant and cell samples were collected at 36 and 48 hpi to detect PRRSV ORF7 expression (Figure 2C), virus titre (Figure 2D), and PRRSV N expression (Figure 2E). The results showed that SC strongly inhibited PRRSV infection in a dose-dependent manner.

Ribavirin has been reported to significantly inhibit the replication of PRRSV VR-2332 in Marc-145 cells at 200 µM [19]. Therefore, 200 µM ribavirin was used as an anti-PRRSV positive control in Marc-145 cells.

### SC suppresses PRRSV infection and inflammatory cytokine secretion in PAMs

**Figure 3:**
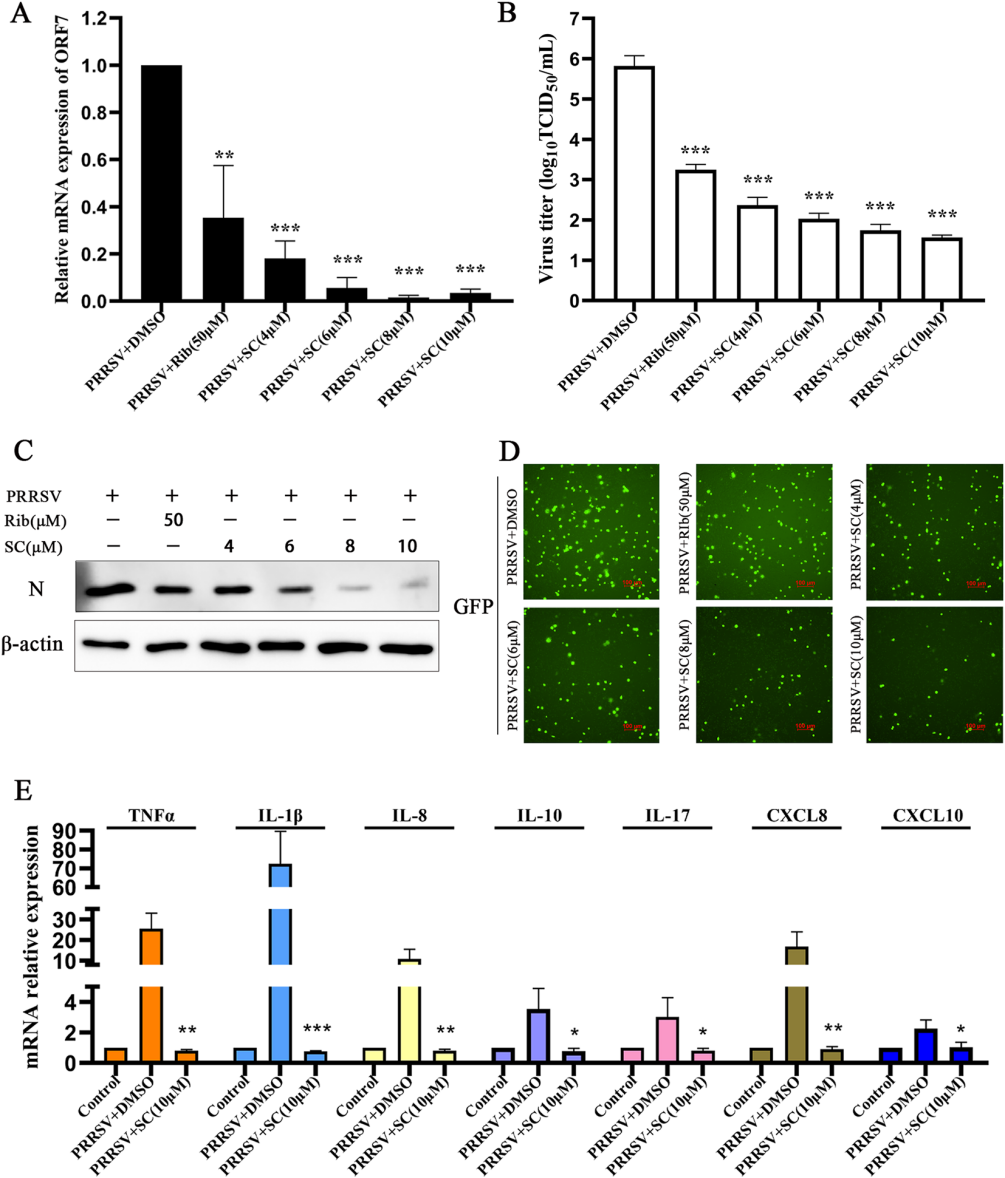
**SC suppresses PRRSV infection and inflammatory cytokine expression in PAMs.** **A**–**C** PAMs were seeded in 12-well plates, cultured for 6 h, infected with PRRSV SD-YL1712 (MOI = 1), and then treated with different concentrations of SC. Supernatants and cells were harvested at 24 hpi to determine PRRSV ORF7 expression, virus titre and PRRSV N expression. **D** PAMs were seeded in 96-well plates, cultured for 6 h, infected with GFP-PRRSV (MOI = 1), and treated with different concentrations of SC. After 24 h of GFP-PRRSV infection, the fluorescence of GFP was measured with a Nikon fluorescence microscope. **E** PAMs were seeded in 12-well plates, cultured for 6 h, infected with PRRSV SD-YL1712 (MOI = 1), and then treated with 10 µM SC. Cells were harvested at 24 hpi to measure inflammatory cytokine levels. *P < 0.05; **P < 0.01; ***P < 0.001 compared with the respective virus control.

To verify the inhibitory effect of SC on PRRSV infection in PAMs, we first examined the toxicity of 10 µM SC to PAMs (Figure 6B) and then determined PRRSV ORF7 expression, viral titre, and PRRSV N expression. As shown in Figures 3A–C, SC significantly reduced the PRRSV ORF7 expression level, viral titre, and N protein expression level in PAMs in a dose-dependent manner. Then, PAMs were infected with the GFP-PRRSV strain to detect the effect of SC on virus replication. The results showed that SC treatment strongly inhibited the replication of the GFP-PRRSV strain (Figure 3D). Next, PAMs were infected with PRRSV SD-YL1712 and treated with SC to detect the expression levels of inflammatory cytokines. As shown in Figure 3E, the levels of inflammatory cytokines in PAMs increased sharply after PRRSV infection, but SC treatment alleviated this phenomenon. These results indicate that SC significantly inhibits PRRSV replication and reduces the expression levels of inflammatory cytokines in PAMs.

It has been reported that ribavirin can markedly suppress PRRSV replication in PAMs at 50 µM [20]. Therefore, we used 50 µM ribavirin as an anti-PRRSV positive control in PAMs.

**Figure 4:**
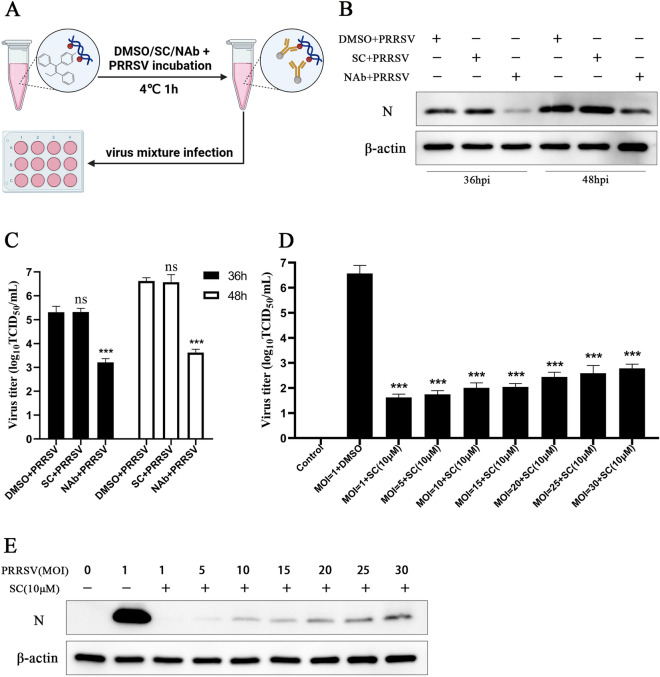
**SC does not interact with PRRSV directly.** **A**–**C** Schematic diagram of the interaction between SC and PRRSV. Ten microlitres of 1 mM SC solution was added to 990 µL of PRRSV original solution. Ten microlitres of DMSO was added to the negative control group, and 10 µL of neutralization antibody was added to the positive control group. After incubation at 4 °C for 1 h, Marc-145 cells were infected with 2 µL of the virus mixture, and supernatant and cell samples were collected at 36 and 48 hpi to measure PRRSV N expression levels and virus titres. **D**, **E** Marc-145 cells were infected with PRRSV at MOIs ranging from 1 to 30, and supernatant and cell samples were collected at 36 hpi to detect the virus titre and PRRSV N expression level. *P < 0.05; **P < 0.01; ***P < 0.001 compared with the respective virus control.

To determine whether SC can interact directly with PRRSV in vitro, the virus was incubated with 10 µM SC at 4 °C for 1 h, and the virus mixture was used to infect Marc-145 cells (Figure 4A). The supernatant and cells were collected at 36 hpi and 48 hpi to detect the viral titre and PRRSV N expression level. As shown in Figures 4B and C, SC did not interact with PRRSV directly, and treatment with the neutralization antibody significantly reduced the viral titre and N expression level. Next, Marc-145 cells were infected with PRRSV at MOIs ranging from 1 to 30 and treated with SC (10 µM), and the viral titre and PRRSV N expression level were measured at 36 hpi. The results showed that SC markedly reduced the virus titre (Figure 4D) and N expression level (Figure 4E) at all infective doses.

### The preventive treatment mode is the most effective way

**Figure 5:**
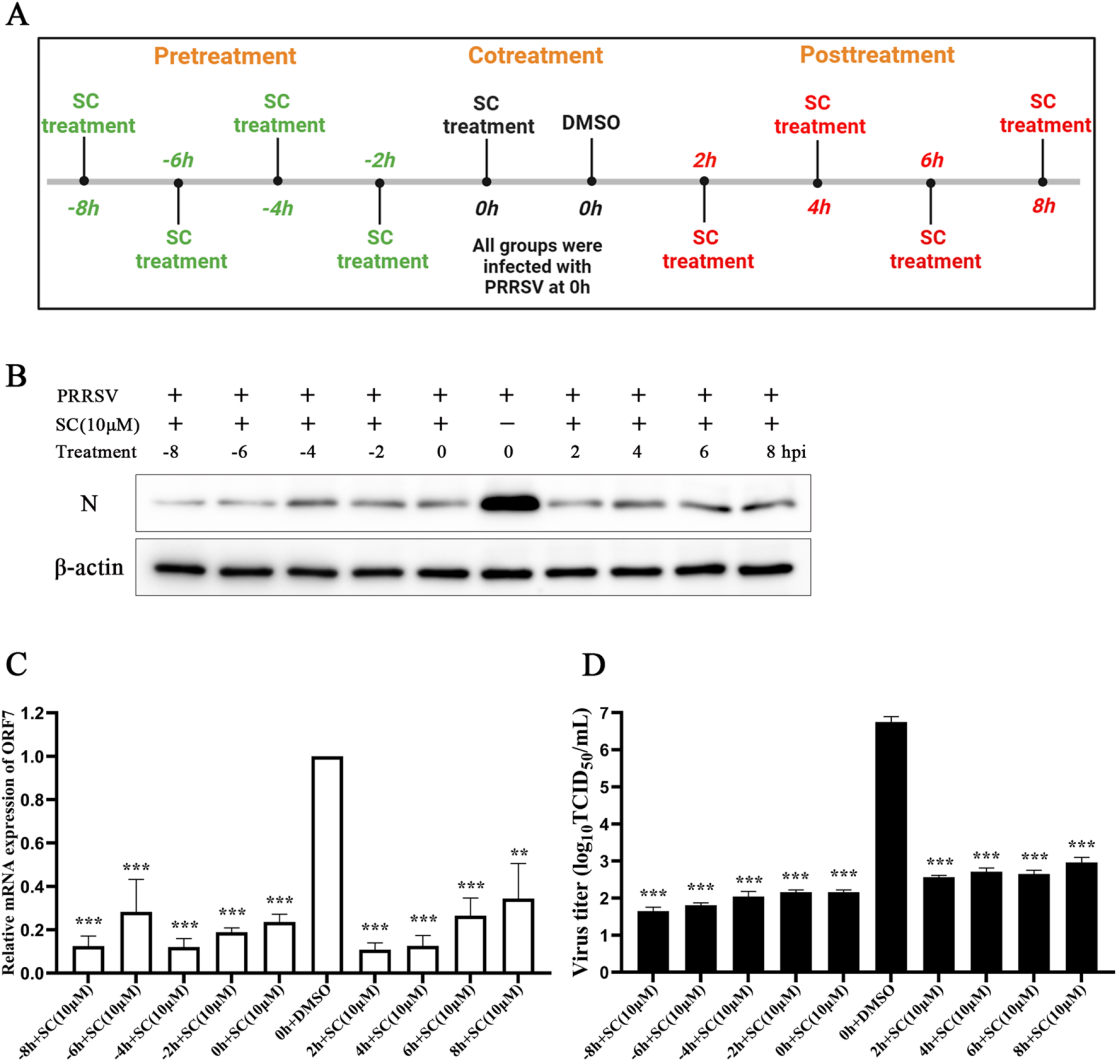
**SC inhibits PRRSV infection after different modes of treatment.** SC was added before (pre-treatment), during (co-treatment), or after (post-treatment) PRRSV SD-YL1712 infection (MOI = 1). The pre-treatment groups were inoculated with PRRSV 8, 6, 4, or 2 h after the addition of SC. The co-treatment group was infected with PRRSV and immediately treated with 10 µM SC. In the post-treatment group, cells were infected with PRRSV first, and SC was added at 2, 4, 6, or 8 h after infection. Supernatants and cells were harvested at 24 hpi for RT-qPCR, Western blotting, and viral titre assays. **A** Diagram of the timeline for the treatment mode experiments. **B** PRRSV N expression level. **C** PRRSV ORF7 expression level. **D** Supernatant virus titre. *P < 0.05; **P < 0.01; ***P < 0.001 compared with the respective virus control.

Cells were treated according to the timeline to explore the antiviral effect of SC administered via different treatment modalities, and a sampling time point was set for every 2 h. SC was added to the cell cultures before (pre-treatment), during (co-treatment), and after (post-treatment) PRRSV infection (Figure 5A). At 24 hpi, supernatant and cell samples were collected to detect PRRSV N and ORF7 expression and virus titre. As shown in Figure 5B, the expression level of PRRSV N was the lowest after SC pre-treatment for 8 h and gradually increased over time, while the inhibitory effect on viral N expression at 8 h in the post-treatment group was greatly reduced. The expression level of PRRSV ORF7 (Figure 5C) and virus titre (Figure 5D) results show that pre-treatment with SC has the best effect on inhibiting viral replication, followed by co-treatment, and post-treatment is the least effective. These results indicate that all treatment modes can significantly suppress PRRSV replication, but preventive treatment is the most effective.

### SC inhibits the replication of different PRRSV strains and multiple viruses

**Figure 6:**
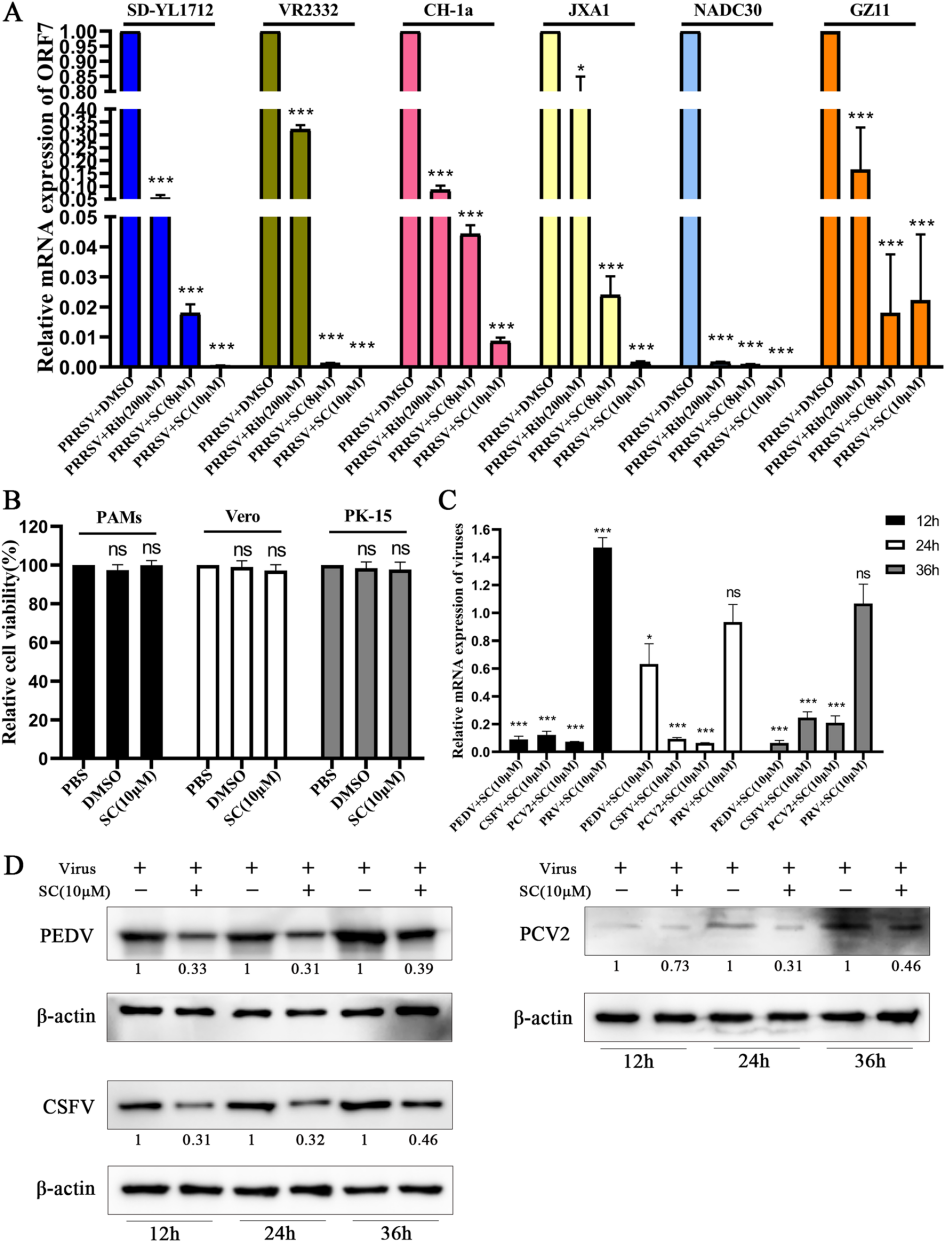
**SC inhibits the replication of different PRRSV strains and multiple other viruses.** **A** Marc-145 cells were seeded in 12-well plates, cultured for 12 h, and infected with different PRRSV strains (SD-YL1712, VR-2332, CH-1a, JXA1, NADC30, and type 1 PRRSV GZ11). Thirty-six hours after PRRSV infection, cell samples were collected to measure the PRRSV ORF7 expression level. **B** Forty-eight hours after treatment with 10 µM SC, the toxicity of 10 µM SC to PAMs, Vero cells, and PK-15 cells was detected by CCK-8 assay. PBS was used as a negative control, and the cell viability in this group was set to 100%. **C**, **D** Vero cells were infected with PEDV, and PK-15 cells were infected with PCV2, CSFV, and PRV (MOI = 1). Cells were harvested at 12, 24, and 36 hpi to determine the expression levels of viral genes and viral proteins. *P < 0.05; **P < 0.01; ***P < 0.001 compared with the respective virus control.

To verify the inhibitory effect of SC on different PRRSV strains, Marc-145 cells were infected with different PRRSV strains (SD-YL1712, VR-2332, CH-1a, JXA1, NADC30 and PRRSV type 1 strain GZ-11) and treated with different concentrations of SC. The expression levels of PRRSV ORF7 were measured at 24 hpi, and the results indicated that SC treatment significantly reduced the replication of different PRRSV strains (Figure 6A).

Next, to evaluate the inhibitory effect of SC on the other four major porcine viruses, we first measured the toxicity of 10 µM SC to Vero cells and PK-15 cells by CCK-8 assays and found that SC was not toxic to these cells (Figure 6B). Then, Vero cells were infected with porcine epidemic diarrhoea virus (PEDV), and PK-15 cells were infected with porcine circovirus 2 (PCV2), classical swine fever virus (CSFV), and pseudorabies virus (PRV), and all cells were treated with 10 µM SC. As shown in Figure 6C, the viral gene expression of PEDV, PCV2, and CSFV was decreased to various degrees by 10 µM SC, but SC did not inhibit PRV infection. Then, we examined whether the PEDV, PCV2, and CSFV protein expression levels were reduced. The results showed that 10 µM SC suppressed the expression levels of the PEDV N protein, PCV2 Cap protein and CSFV E2 protein (Figure 6D). These results suggest that SC alleviates PEDV, PCV2, and CSFV infection but not PRV infection.

### SC suppresses the activation of NF-κB to inhibit PRRSV infection

**Figure 7:**
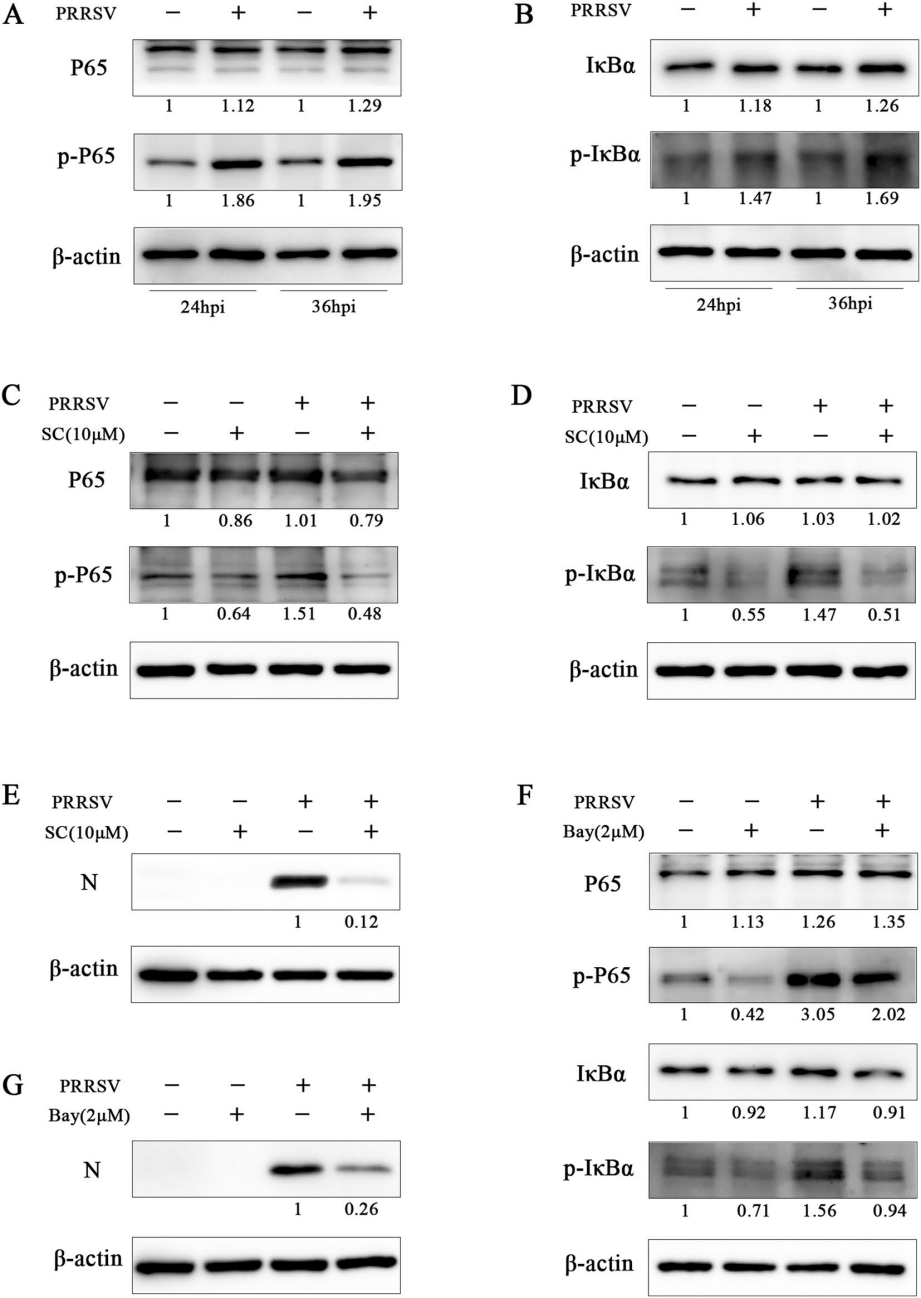
**SC suppresses the activation of NF-κB to inhibit PRRSV infection.** **A**, **B** Marc-145 cells were seeded in 12-well plates and cultured for 12 h. The cells were infected with PRRSV SD-YL1712 (MOI = 1), and cells were collected at 24 and 36 hpi for Western blotting to determine the expression levels of P65, p-P65, IκBα, and p-IκBα. **C**–**E** Marc-145 cells in 12-well plates were infected with PRRSV SD-YL1712 (MOI = 1) and then treated with 10 µM SC. Cells were harvested at 36 hpi for Western blotting to determine the expression levels of P65, p-P65, IκBα, p-IκBα, and PRRSV N. **F**, **G** Marc-145 cells were seeded in 12-well plates, cultured for 12 h, infected with PRRSV SD-YL1712 (MOI = 1), and then treated with 2 µM Bay11-7082. At 36 hpi, cells were collected and analysed to determine the expression levels of P65, p-P65, IκBα, p-IκBα, and N by Western blotting.

Since pre-treatment is a more effective treatment mode, it is reasonable to assume that SC inhibits PRRSV proliferation by limiting the functions of the intracellular components that promote viral replication. In addition, SC treatment reduced the expression levels of inflammatory cytokines in PAMs (Figure 3E). Therefore, we evaluated the effect of SC on the NF-κB signalling pathway. The results showed that the expression of p-P65 and p-IκBα (Figures 7A and B) was significantly increased after PRRSV infection, but SC treatment could significantly reverse this trend (Figures 7C and D), and the PRRSV N expression level was also significantly decreased (Figure 7E). Then, we used an NF-κB inhibitor (Bay11-7082) to treat Marc-145 cells after PRRSV infection. The results showed that the expression levels of p-P65 and p-IκBα were significantly reduced after treatment with the inhibitor (Figure 7F), and the PRRSV N expression level was also inhibited (Figure 7G). These results indicate that PRRSV infection activates the NF-κB signalling pathway and that SC suppresses PRRSV infection by inhibiting activation of the NF-κB signalling pathway.

### SC inhibits the NF-κB signalling pathway by promoting TRAF2 expression

**Figure 8:**
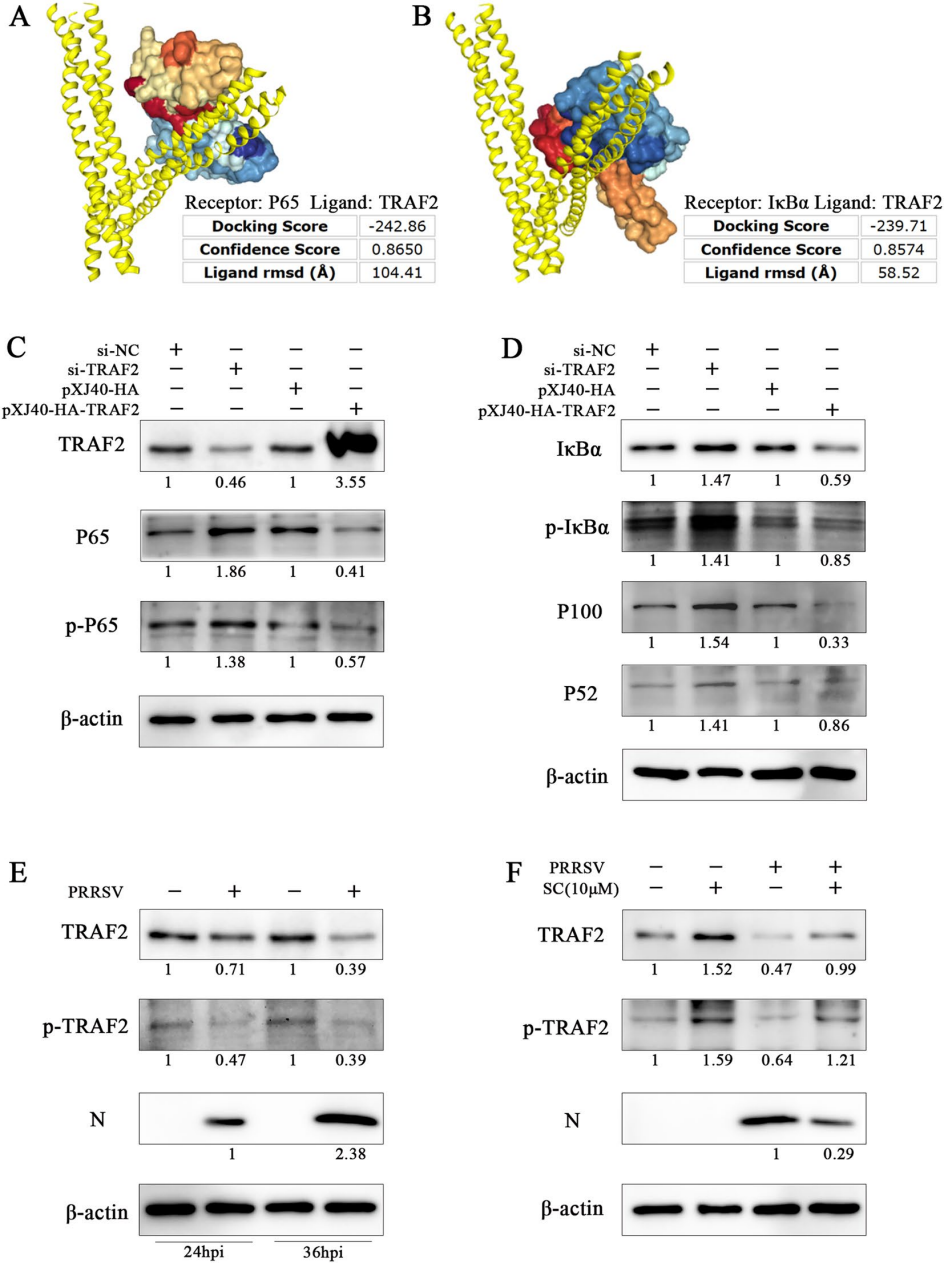
**SC inhibits the NF-κB signalling pathway by promoting TRAF2 expression.** **A**, **B** The interaction between TRAF2 and P65 and between TRAF2 and IκBα was predicted by the HDOCK server online. **C**, **D** Marc-145 cells were seeded in 12-well plates and transfected with siTRAF2 (50 nM), siNC (negative control), pXJ40-HA-TRAF2, or vector. Cells were harvested at 36 hpi after transfection to detect the expression levels of TRAF2, P65, p-P65, IκBα, p-IκBα, P100 and P52. **E** Marc-145 cells were seeded in 12-well plates and cultured for 12 h. The cells were infected with PRRSV SD-YL1712 (MOI = 1) and collected at 24 and 36 hpi for Western blotting to determine the expression levels of TRAF2 and p-TRAF2. **F** Marc-145 cells in 12-well plates were infected with PRRSV SD-YL1712 (MOI = 1) and then treated with 10 µM SC. Cells were harvested at 36 hpi for Western blotting to determine the expression levels of TRAF2, p-TRAF2, and PRRSV N.

To elucidate the molecular mechanism by which SC inhibits the NF-κB signalling pathway, we investigated the possible upstream regulatory molecules of NF-κB. The HDOCK server is a protein‒protein and protein‒DNA/RNA docking online tool based on a hybrid algorithm of template-based modelling and ab initio free docking data [21, 22]. We used this online tool to predict the interaction between TRAF2 and NF-κB, and the results showed that TRAF2 could bind to P65 and IκBα with binding energies of − 242.86 kcal/mol and − 239.71 kcal/mol, respectively, with corresponding confidence levels of 0.8650 and 0.8574 (Figures 8A and B). Next, we further verified the effect of TRAF2 on the NF-κB signalling pathway. As shown in Figures 8C and D, knockdown of TRAF2 significantly promoted the expression of P65, p-P65, IκBα, p-IκBα, P100 and P52. Ectopic expression of TRAF2 remarkably inhibited the expression of these proteins. These results demonstrate that TRAF2 is a major negative regulator of NF-κB. Next, we verified the effect of SC on TRAF2 expression. We first detected the effect of PRRSV infection on TRAF2 expression and then treated PRRSV-infected Marc-145 cells with 10 µM SC. As shown in Figures 8E and F, PRRSV infection decreased TRAF2 expression, while SC treatment significantly reversed this trend by increasing TRAF2 and p-TRAF2 expression. These results suggest that SC inhibits PRRSV replication by promoting TRAF2 expression to suppress activation of the NF-κB signalling pathway.

### PRRSV Nsp10 interacts with TRAF2 to decrease its expression

**Figure 9:**
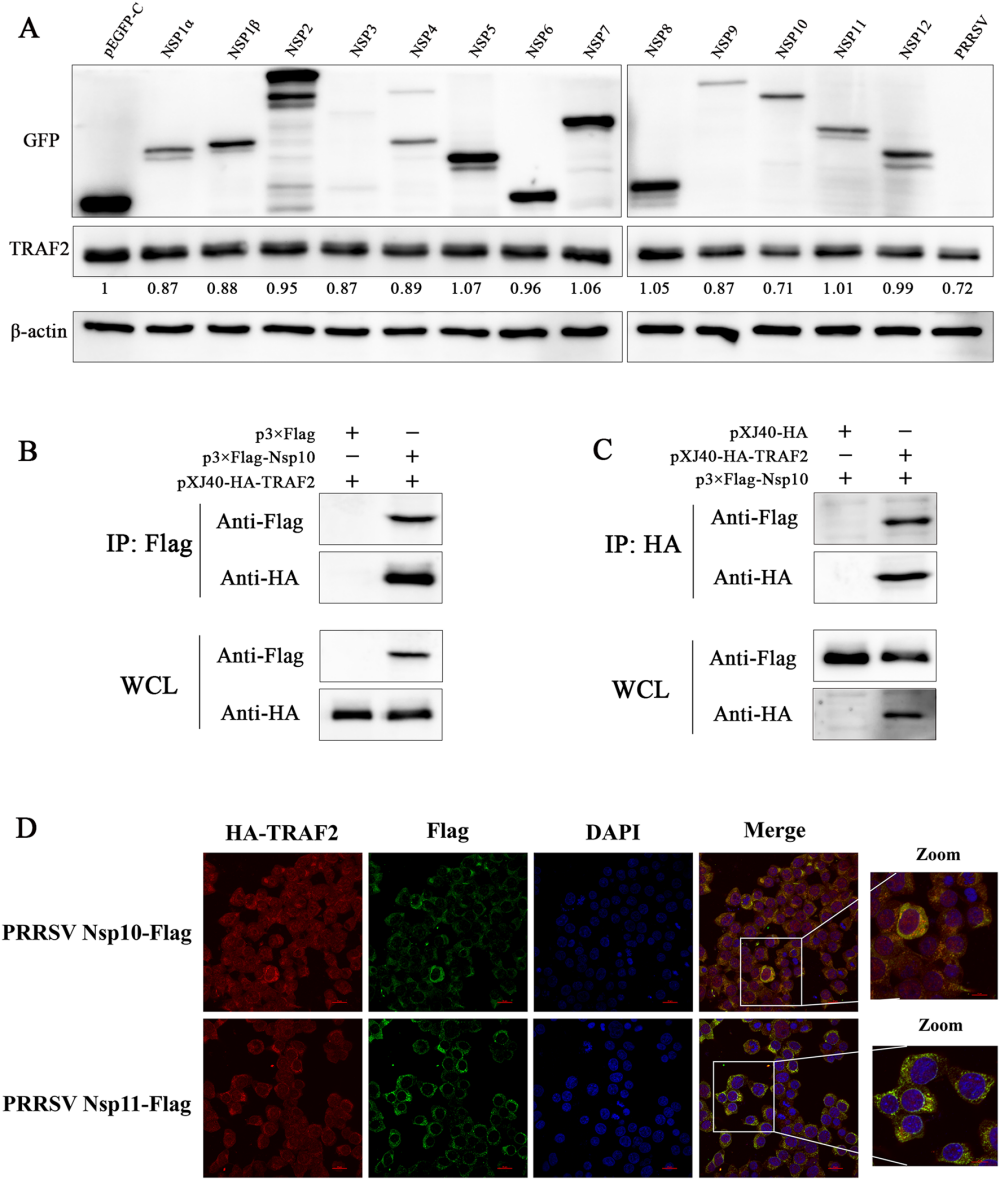
**PRRSV Nsp10 interacts with TRAF2 and decreases its expression.** **A** CRL-2843 cells were transfected with pEGFP-C-PRRSV nonstructural proteins for 36 h and then harvested to determine the expression of pEGFP-C-Nsps and TRAF2. **B** HEK-293T cells were cotransfected with p3×Flag-Nsp10 and pXJ40-HA-TRAF2. After 36 h, the cells were collected, and Co-IP was used to determine the interaction between the Nsp10 protein and TRAF2. **C** HEK-293T cells were transfected with pXJ40-HA-TRAF2 and p3×Flag-Nsp10. After 36 h, the cells were collected to determine the reverse Co-IP interaction between TRAF2 and Nsp10. **D** HEK-293T cells were cotransfected with the p3×Flag-Nsp10 and pXJ40-HA-TRAF2 or p3×Flag-Nsp11 and pXJ40-HA-TRAF2 proteins. After 24 h, the cells were stained with anti-Flag and anti-HA antibodies to determine the position of Nsp10 (green) relative to TRAF2 (red), and images were captured by confocal microscopy.

Next, how PRRSV downregulates the expression of TRAF2 to activate the NF-κB signalling pathway is illustrated. Since PRRSV nonstructural proteins are needed for viral genome replication, we transfected the PRRSV nonstructural proteins Nsp1α to Nsp12 into CRL-2843 cells, which are immortalized PAMs. As shown in Figure 9A, compared with the empty vector pEGFP-C, TRAF2 expression was significantly decreased in the Nsp10 transfection group, indicating that Nsp10 played the most important role in downregulating TRAF2 expression. Then, Nsp10 and TRAF2 were cotransfected into HEK-293T cells and cultured for 36 h to collect cell lysates for the coimmunoprecipitation (Co-IP) assay. The results showed that pXJ40-HA-TRAF2 interacted with p3×Flag-Nsp10 but did not interact with the p3×Flag empty vector (Figure 9B). In the reverse Co-IP experiment, p3×Flag-Nsp10 interacted with pXJ40-HA-TRAF2 but did not interact with the pXJ40-HA empty vector (Figure 9C). Next, we further confirmed the colocalization of Nsp10 and TRAF2 by fluorescence confocal microscopy and found that TRAF2 did not colocalize with Nsp11 (Figure 9D). These results demonstrated the specificity of the interaction between Nsp10 and TRAF2.

## Discussion

PRRS causes tremendous economic losses to the pig industry worldwide, with annual losses of approximately US $660 million in the United States and EUR 75 700 to EUR 650 000 in Europe [23, 24]. PRRS causes serious secondary infections, immunosuppression, and persistent damage, greatly harming the pig industry. In addition, the extensive use of antibiotics poses a potential threat to public health security. Therefore, we must develop effective and safe natural small molecule compounds against PRRSV infection from the perspective of the host to find efficient treatment options to supplement the existing prevention and control measures.

**Figure 10:**
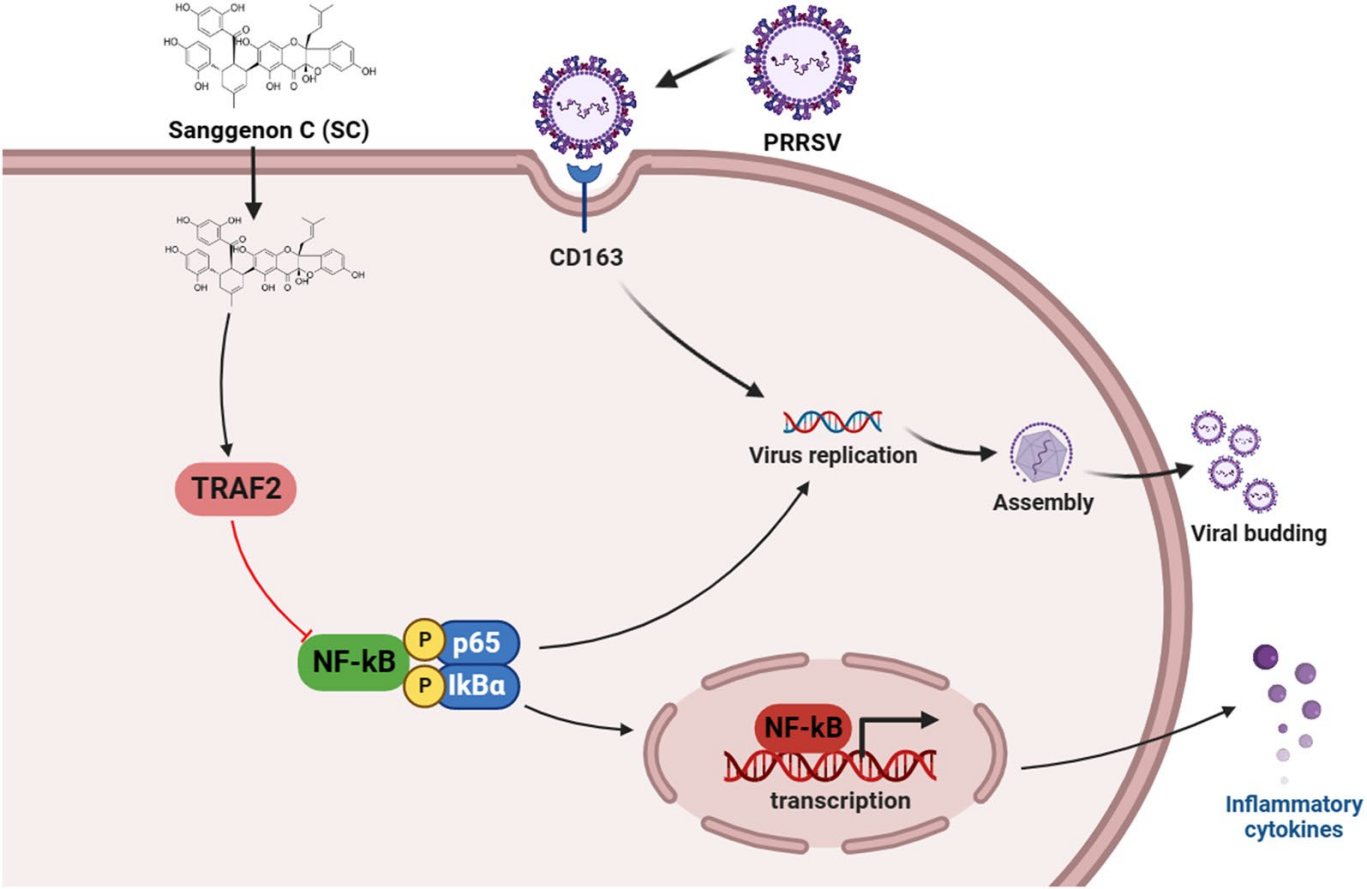
**SC inhibits PRRSV infection by regulating the TRAF2/NF-κB signalling pathway.** PRRSV infection promotes activation of the NF-κB signalling pathway via inhibition of TRAF2 expression, thus promoting self-replication. SC suppresses activation of the NF-κB signalling pathway by promoting the expression of TRAF2, thereby inhibiting PRRSV replication and the expression of inflammatory cytokines. The schematic was drawn using the Biorender website.

Sanggenon C is a flavanone Diels–Alder adduct isolated from the root bark of mulberry that not only has anti-inflammatory [25] and antiosteoporotic [26] properties but also inhibits the activity of pancreatic lipase [27]. Studies have reported that Sanggenon C inhibits the growth activities of prostate cancer cells by inducing cell cycle arrest and promoting apoptosis [28]. SC suppresses mitochondrial fission and induces apoptosis in gastric cancer cells through the ERK signalling pathway [10], alleviates mutagenic and inflammatory effects in intestinal cells induced by intestinal microbial nitroreductase [29] and *E. coli* β-glucuronidase [30], and regulates the production of reactive oxygen species and nitric oxide to induce colon cancer cell apoptosis [31]. However, the mechanism of action of SC was fully elucidated in these studies, and no relevant studies have reported its antiviral activity. This study demonstrated for the first time that SC effectively inhibits PRRSV, PEDV, PCV2, and CSFV infection at a concentration of 10 µM and found that PRRSV Nsp10 activated the NF-κB signalling pathway by inhibiting TRAF2 expression. Furthermore, we fully demonstrated that SC inhibits the activation of the NF-κB signalling pathway by promoting TRAF2 expression, thereby reducing PRRSV infection (Figure 10).

TRAF2 is a member of the tumour necrosis factor receptor-associated factor (TRAF) family of molecules, which not only induces innate immunity, inflammatory cytokines, and factors related to cell survival but also regulates endoplasmic reticulum (ER) stress signalling, autophagy, cell death, and various cancer-related cellular processes [32]. Studies have reported that TRAF2 plays a negative regulatory role in the expression of TLR-stimulated proinflammatory cytokines and inhibits the occurrence of colitis by reducing the expression of LPS-, poly(I:C)- and IL-1β-induced proinflammatory cytokines [33]. In addition, TRAF2 can regulate the occurrence and development of inflammation by protecting intestinal epithelial cells from TNFα-induced apoptosis and thereby inhibiting epithelial inflammation [34]. However, the molecular mechanism by which TRAF2 inhibits inflammation in PRRSV infection has not been fully elucidated, and the mechanism by which the virus downregulates TRAF2 expression also needs further study.

Nuclear factor-κB (NF-κB) comprises five families of transcription factors involved in various cellular processes and plays a pivotal role in mediating inflammatory responses [35]. It has been reported that activation of NF-κB is involved in the replication processes of a variety of viruses, such as dengue virus [36], classical swine fever virus [37], and African swine fever virus [38]. From these data, inhibiting NF-κB activation is a feasible strategy to prevent viral replication [39]. Recently, many plant extracts including compounds such as flavonoids, lignans, alkaloids, polyphenols, and their derived compounds have been evaluated as potential inhibitors of the NF-κB signalling pathway [40], which has guided the development and application of natural antiviral compounds.

Our study found that the natural compound Sanggenon C inhibits the NF-κB signalling pathway by promoting TRAF2 expression, thereby alleviating PRRSV infection. Moreover, SC showed a great protective effect against infection by different PRRSV strains and could limit the replication of PRRSV at an MOI of 30. Pre-treatment was the most effective treatment mode, which is possibly related to the time needed for SC to regulate the TRAF2/NF-κB signalling pathway. In addition, Sanggenon C can also inhibit PEDV, PCV2, and CSFV replication but not PRV replication. These data increase the possibility of using SC as an effective drug against multiple viral infections in the pig industry. In summary, we identified that Sanggenon C alleviates PRRSV infection by promoting TRAF2 expression to inhibit the NF-κB signalling pathway. These results suggest that SC is a potential and promising candidate for controlling PRRS.

## Data Availability

All data generated or analysed during this study are included in this published article.
